# Impedance Imaging of Cells and Tissues: Design and Applications

**DOI:** 10.34133/2022/9857485

**Published:** 2022-06-01

**Authors:** Raziyeh Bounik, Fernando Cardes, Hasan Ulusan, Mario M. Modena, Andreas Hierlemann

**Affiliations:** ETH Zürich, Department of Biosystems Science and Engineering, Basel, Switzerland

## Abstract

Due to their label-free and noninvasive nature, impedance measurements have attracted increasing interest in biological research. Advances in microfabrication and integrated-circuit technology have opened a route to using large-scale microelectrode arrays for real-time, high-spatiotemporal-resolution impedance measurements of biological samples. In this review, we discuss different methods and applications of measuring impedance for cell and tissue analysis with a focus on impedance imaging with microelectrode arrays in *in vitro* applications. We first introduce how electrode configurations and the frequency range of the impedance analysis determine the information that can be extracted. We then delve into relevant circuit topologies that can be used to implement impedance measurements and their characteristic features, such as resolution and data-acquisition time. Afterwards, we detail design considerations for the implementation of new impedance-imaging devices. We conclude by discussing future fields of application of impedance imaging in biomedical research, in particular applications where optical imaging is not possible, such as monitoring of *ex vivo* tissue slices or microelectrode-based brain implants.

## 1. Introduction

Recent progress in *in vitro* cellular and molecular analysis has greatly advanced our understanding of human physiology in healthy and diseased states. Owing to their label-free and noninvasive nature, impedance-based detection methods have been used for quantitative long-term characterization of live cells and tissues. In comparison to other routine analysis methods, such as optical imaging, impedance measurements typically require less costly equipment, the respective setups can be miniaturized, and experiments can be parallelized [1, 2].

First reports on the use of impedance-based detection methods in biology date back to the 1920s, when impedance measurements were used to characterize membrane capacitance and resistance of blood cells [3, 4]. Technical improvements and the establishment of theoretical models have then enabled the use of impedance methods for the analysis of cell volumes in suspension [5], for discerning different cell populations [5], and for monitoring cellular adhesion and growth [6–8]. The development of high-density microelectrode arrays (MEAs) [9] and their use for impedance analysis enabled high-sensitivity and spatially highly resolved measurements at subcellular resolution [10–13]. The label-free and high-resolution nature of impedance detection offered by high-density MEAs has enabled to produce 2D impedance images of biological samples to, e.g., monitor how cell attachment and mobility of adherent cells are affected by drug treatment [11] or to recognize the spatial organization of cells in live, *ex vivo* brain slices [12].

In this review article, we first present different examples of how impedance measurements and impedance imaging is applied in neuroscience and biomedical research. We discuss different methods that have been introduced for the characterization of cell and tissue models *in vitro*, with a particular focus on methods and approaches for two- and three-dimensional (2D and 3D) impedance-based imaging, and we present examples of impedance imaging in biomedical research. We then discuss the different detection methods that are used for impedance analysis as well as related circuitry implementations and discuss the advantages and limitations of the different approaches. Afterwards, we summarize the design options that need to be considered during the conceptualization of impedance-based MEA sensors for different applications. Finally, we end the review with an outlook on trends in developing MEAs for impedance-based imaging of biological entities.

## 2. Impedance Measurements: Methods and Applications

### 2.1. Impedance Measurements and Impedance Model of Cells and Tissues

Impedance extends the concept of Ohm’s resistance to alternating-current (AC) circuits and features both, magnitude and phase, unlike Ohmic resistance, which has only magnitude. Impedance is a complex number with the same unit as resistance, for which the SI unit is Ohm (*Ω*), its symbol is usually Z. In analogy to Ohms law for directed current (DC), the impedance (Z) is the ratio of the electrical potential difference (V), applied to a conductor, and the resulting current (I) through it (Equation (1)). Impedance can, therefore, be expressed as the combination of a resistance (R, the real component) and a reactance (X, the imaginary component):

(1)Z=Vej θVIej θI=ZejθZ=R+jX,

where $\left|∙\right|$ and $\theta_{\left(∙\right)}\;$ represent the modulus and phase of the respective complex number, and j is the imaginary unit.

**Figure 1:**
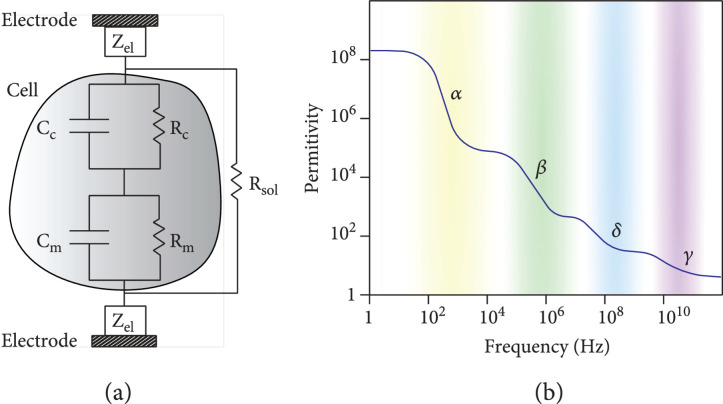
(a) Electrical equivalent-circuit model of a cell in suspension between a pair of electrodes. The overall measured impedance includes three contributions: the cell impedance, which consists of the cytoplasm and membrane capacitive and resistive contributions ($C_{c}$, $R_{c}$ and $C_{m}$, $R_{m}$), the electrode impedances ($Z_{\text{el}}$), and the medium resistance ($R_{\text{sol}}$). (b) Idealized spectrum of the relative permittivity of cells and tissues, showing four main dispersion regions [18, 19]; for details, see text.

Impedance sensing is based on measuring the absolute value or relative change in the impedance of a cell or tissue to extract information on sample properties. Impedance measurements can be carried out at a specific frequency or over a broad frequency range, the latter being termed electrical impedance spectroscopy (EIS). Figure 1(a) shows a simplified electrical equivalent-circuit model of a single cell between a pair of electrodes. The overall impedance of the system includes three main components: (i) the cell impedance, which consists of membrane contributions ($C_{m}$ and $R_{m}$ in parallel) and cytoplasm contributions ($R_{c}$ and $C_{c}$ in parallel); the cell equivalent circuit is known as the single-shell model [14]; as the resistive impedance component of the cell membrane and the capacitive impedance component of the cytoplasm are usually orders of magnitudes larger than the capacitive component of the cell membrane and the resistive component of the cytoplasm, the contributions of these electrical equivalent-circuit components are typically neglected in the electrical equivalent-circuit models [15]; (ii) the impedance of the electrode-electrolyte interfaces, $\;Z_{\text{el}}$, which consists of the double layer capacitances (main contribution for polarizable electrodes, such as platinum (Pt) or gold (Au) electrodes) in parallel to the charge-transfer resistances (main contribution for nonpolarizable electrodes, such as silver/silver chloride (Ag/AgCl) electrodes) [16]; (iii) the solution resistance $R_{\text{sol}}$ [16].

When an electric field is applied to a dielectric material, such as a cell membrane, the field causes a polarization of the material. The polarization response of a dielectric material to an externally applied field is characterized by its permittivity, which is associated with its ability to store energy. As the energy of an applied electric field can either be dissipated or stored, the impedance of a dielectric material is directly related to its permittivity and can be expressed as [17]

(2)z=σ−jωϵ0ϵσ2+ωϵ0ϵ2,

where σ is the characteristic conductivity at DC, ω is the radial frequency of the applied electric field, $\epsilon_{0}$ is the permittivity of vacuum, and ϵ is the relative permittivity of the respective dielectric material. Typically, the relative permittivity of a material is frequency-dependent and tends to fall as the frequency of the applied field increases [18]. For cells and tissues in solution, the relative permittivity extends over four principal dispersion regions, i.e., frequency ranges in which the relative permittivity features large variations (Figure 1(b)): the *α*-dispersion, in the ~100 Hz-10 kHz region, which is associated with ion diffusion across the cell surface; the *β*-dispersion, in the sub-MHz–10 MHz range, which is related to charge accumulation at the cellular membrane; the *δ*-dispersion in the sub-GHz regime, in which rotation of macromolecular side-chains takes place; the *γ*-dispersion in the ~10 GHz regime, where dipolar rotation of water molecules is dominant [18, 19]. Therefore, measurements of cell and tissue impedance at different frequencies can be used to investigate different phenomena. At low frequencies, in the *α*-dispersion frequency range, the cell impedance is extremely high so that impedance measurements provide information on the cell or tissue volume, which obviates current conduction. Therefore, measurements in this frequency range can be used to estimate cell or tissue volume or size. Upon increasing the frequency, information on cell and tissue membranes can be extracted, as the cell membrane polarization is in the *β*-dispersion range. Higher frequencies, in the sub-GHz and GHz regime, can provide information on the water content or protein concentrations within cells [20, 21]. However, these frequency ranges are rarely used for investigating cells and tissues, as measurements at such high frequencies require careful electronic design, and as cell/tissue impedance contributions cannot be easily separated from, e.g., medium contributions, due to the dominant effect of dielectric relaxation of water in the GHz regime [22].

### 2.2. Impedance Measurement Methods

Impedance measurements are typically carried out by applying an electrical stimulus to the sample of interest and by measuring the sample response as a function of the frequency of the applied signal. Two different implementations are possible: a known test voltage is applied to the sample, and the resulting current is measured, or, alternatively, a test current is injected, and the resulting voltage drop across the sample is detected. The two approaches are conceptually equivalent from a theoretical point of view and are based on the generalized Ohm’s law (Equation (1)). However, the respective implementations require different electronic circuitries and may feature different susceptibility to unwanted effects during sample characterization.

The power and frequency of the stimulation signal are chosen depending on the composition of the sample, the measurement features of interest, and the limitations of the instrumentation. Signal power must be high enough to produce a response that can be detected by the readout electronics, however, without saturating the amplifiers. Moreover, large stimulation currents can damage a sample, as it is intentionally done in electronic wound-healing assays [6], while high voltages can cause undesired electrochemical reactions in an aqueous phase, such as electrolysis. Stimulations can be carried out by using sinusoidal waves, the signal power of which is concentrated at one specific frequency, or the stimulus can consist of a multifrequency signal, where the signal power is spread across a broader frequency spectrum.

Finally, impedance detection can also be carried out in an indirect way, for example, through impedance-to-frequency conversion. This conversion method and its implementation in integrated systems will be discussed in more detail in Section 3.5.

#### 2.2.1. Stimulation Frequency Selection

**Figure 2:**
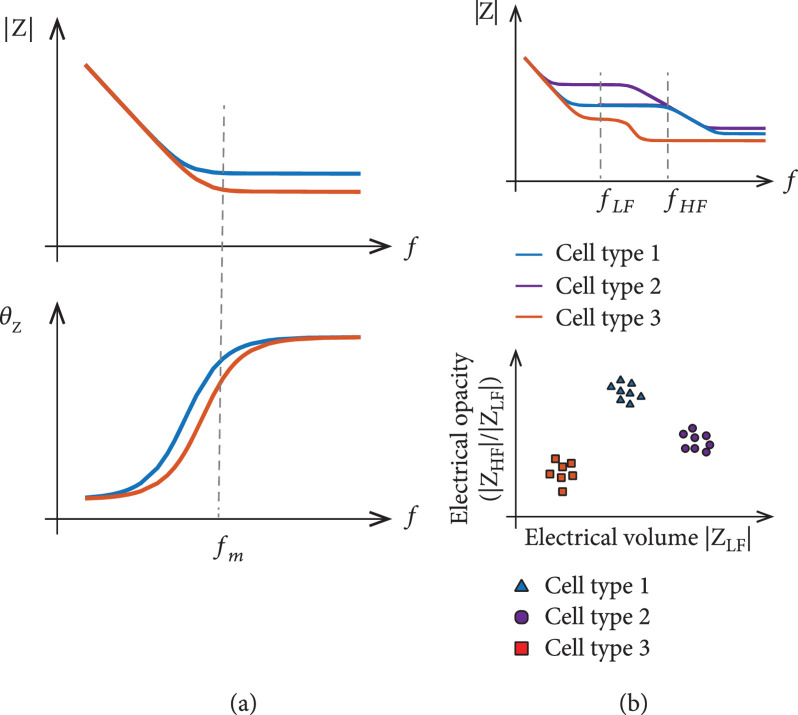
Examples of impedance measurements at different frequencies. (a) Illustration of the impedance of two samples, the capacitive contribution of which is dominant at low frequencies while the resistive component is dominating at high frequencies. A single measurement at $f_{m}$ allows for detection of resistance changes of the sample. Note that phase variations and magnitude variations manifest at different frequencies. (b) Example of cell classification using two-frequency impedance measurements. Low frequency ($f_{\text{LF}}$) impedance provides information on the cell size, while the ratio of high-frequency ($f_{\text{HF}}$) and low-frequency impedance yields the so-called electrical opacity.

Single-frequency measurements are commonly used when the target feature is known to be observable at a specific frequency (Figure 2(a)). For example, cellular attachment to an electrode surface can be detected at 1 kHz [23], cellular micromotions across electrodes can be observed using a 4 kHz sinusoidal signal [7], or parasite motility *in vitro* can be detected with a 500 kHz signal [24]. Single-frequency monitoring offers significant advantages with respect to hardware, since all information is contained in a narrow-bandwidth signal that can be measured and separated from out-of-band noise using, for example, lock-in amplifiers.

Combining multiple measurements at different frequencies allows for investigation of more complex features. This approach is often used to acquire information on cell membrane integrity in reference to cell size by determining the ratio of impedances measured at high (cell membrane characteristics) and low (cell size) frequencies (Figure 2(b)), the so-called electrical opacity [25, 26], and to differentiate different cell populations in mixed samples [27]. Simultaneous multifrequency monitoring requires either hardware that can independently generate and process signals at each frequency [28] or sequentially measure the sample responses at different frequencies, however, at the cost of increased acquisition time [29].

In EIS, impedance characteristics are measured at multiple frequencies across several orders of magnitude [30]. These measurements can be used to fit the electrical equivalent circuit of the sample [30, 31] or to use a dedicated mathematical model to extract specific features of interest [32]. EIS measurements can be performed by applying a sinusoidal stimulus, whose frequency is sequentially altered. While this technique is unparalleled in terms of accuracy, the measurement acquisition time can amount to up to several minutes, if there is a large number of frequency steps, especially in the low-frequency range. Faster measurements can be obtained by using more complex stimuli and by distributing the signal power across a wide spectral range, e.g., by applying a chirp stimulus [33], rectangular pulses [34], or white noise [35]. These stimulation methods are sometimes referred to as “time-domain” techniques, as the readout circuit directly records the signal over time, and frequency analysis is carried out during postprocessing, typically by calculating the Fourier transform of the time-domain signal [36]. However, spreading the power of the stimulus over a wide frequency band may result in low power spectral densities, which may reduce the signal-to-noise ratio and renders these techniques less accurate than their narrow-band counterparts.

#### 2.2.2. Electrode Configurations

**Figure 3:**
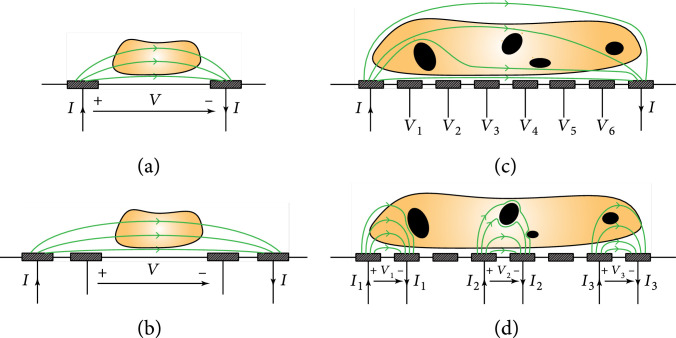
Illustration of impedance measurement methods using a variable number of coplanar electrodes. (a) Two-electrode setup, in which a stimulation current (I) is applied through the same electrodes that are used for voltage measurement (V). Note that voltage stimulation and current measurements are also possible. (b) Four-electrode setup, using one outer pair of electrodes for current stimulation and a second inner pair of electrodes for voltage measurements. (c) EIT uses two (outermost) electrodes of a microelectrode array for current stimulation, while the other electrodes are used to simultaneously measure the voltage at different positions of the sample. (d) Microelectrode array configured for several two-electrode measurements in parallel.

In its simplest form, impedance characterization can be carried out by using a two-electrode configuration (Figure 3(a)). The electrode positions influence the electric-field distribution and how electrical currents flow across the sample. For coplanar electrodes, as shown in Figure 3(a), the electric field decreases with increasing distance to the electrode plane, and most of the current tend to flow along the surface of the electrode plane. Therefore, the measured impedance is significantly altered when a sample is placed on the plane in between the electrodes. In contrast, if the same sample is located far from the electrode plane, it only marginally affects the impedance measured between the electrodes. This position-dependence can be avoided by using facing electrodes, i.e., electrodes placed on opposing planes, which feature uniform electric-field distribution, as in parallel plate capacitors. However, the realization of facing electrodes is more difficult due to a more complex fabrication, and the required alignment of the two electrodes.

Regardless of the electrode configuration, the electrode-electrolyte-interface impedances add in series to the sample impedance, which may obstruct sample characterization at specific frequencies, where electrode contributions dominate. To circumvent this limitation, a multiple-electrode arrangement can be used to reduce or even eliminate the effects of the electrode impedance [29, 37]. In four-electrode measurements, the two outer electrodes (one per terminal) provide the stimulation current, while two inner electrodes sense the voltage drop across the sample (Figure 3(b)). Voltage measurements require that the sensing electrodes do not carry any current so that no voltage drop occurs at the electrodes. The measured signal at the inner electrodes in an arrangement shown in Figure 3(b) reflects the voltage drop across the sample, while the influence of electrode impedance is eliminated. The location of the voltage-sensing electrodes needs to be carefully chosen, in particular for large samples, as the measured voltage drop may otherwise not represent the average voltage drop across the sample.

Electric impedance tomography (EIT) is an impedance-based imaging technique, which relies on measurements of sample impedance at different positions to investigate the internal properties of a sample [38, 39] (Figure 3(c)). A reconstruction algorithm, frequently based on a finite-element model, is used to infer the internal impedance distribution that corresponds to the experimentally determined measurement values. EIT reconstruction is often an ill-posed problem, and algorithms have limitations concerning the complexity of the reconstructed image. Reconstruction typically requires *a priori* information about the expected properties of the sample [40].

Electrode arrays allow for both, multiple two-terminal and multielectrode impedance measurements (Figure 3(d)). Two-terminal measurements can be taken between two electrodes in the array [41, 42], or several electrodes can be grouped to obtain larger pixels to enable multielectrode or differential measurements [43]. Alternatively, one of the terminals can be used as a common reference electrode to perform parallel measurements [10, 44].

#### 2.2.3. Absolute and Relative Measurements

Absolute impedance measurements enable a quantitative characterization of the sample features of interest. However, absolute measurements are strongly dependent on the ability to separate the sample impedance contribution from other effects that may contribute, such as electrode impedance, variation in medium conductivity due to evaporation, and contributions of parasitic circuitry elements. To reduce these unwanted effects on the measurement, differential measurements can be carried out by comparing the sample measurements to reference measurements under the same environmental conditions and acquired with the same readout circuitry. Differential impedance values can either be obtained by determining impedance differences between independent recordings during digital postprocessing [45], for example, by comparing sample impedance recordings to impedance recordings at a reference time point, or by using a differential acquisition scheme including a sample and reference [44, 47].

Calibration is also key to improve the accuracy of impedance measurements. During calibration, the impedance readout circuit is tested with different known samples across the frequency spectrum of interest to verify the response of the system and to enable signal corrections and normalizations during data analysis [29].

### 2.3. Applications of Impedance Measurements

Owing to its label-free and noninvasive nature and its potential for scalability, integration, and automation, impedance spectroscopy has attracted considerable interest for investigating biological and biomedical samples. Here, we will focus on the application of impedance spectroscopy and imaging for *in vitro* studies of cell and tissue models.

#### 2.3.1. Two-Electrode Setups

To obtain a two-dimensional impedance representation or “impedance image” of a sample of interest with a two-electrode setup, the electrode pair needs to be moved across the sample surface. This approach, which is called scanning electrical impedance microscopy (SEIM), was used, e.g., for a morphological characterization of neuronal cells *in vitro* [47, 48]. The topological complexity of cells and tissue models and the long acquisition times of SEIM have, so far, prevented a broader application of this technique for cell and tissue characterization. However, simple two-electrode setups have been widely used to characterize a large variety of biological samples, ranging from single cells to tissue models and multicellular organisms, without acquiring spatial information. We here briefly discuss these systems, as they demonstrate the large variety of information that can be gained through impedance characterization of biological application.

The seminal work of Giaever and Keese in the 1980s and 1990s demonstrated the possibility of monitoring the adherence and micromotion of adherent cells by using a pair of coplanar gold electrodes patterned at the bottom of a cell culture well [7, 49]. The electrode arrangement consisted of a “small” electrode (~10^-2^ mm^2^) and a large counter electrode (~10^2^ mm^2^), so that the overall impedance would be dominated by the electrolytic interface between the small electrode and the medium solution. The presence of cells on top of the small electrode then altered the electrode-medium interface, which could be readily detected by monitoring impedance changes [50]. The technique, which was later termed Electrical Cell-substrate Impedance Sensing (ECIS®), was further developed, and a variety of electrode arrangements have been devised to monitor different parameters of interest, such as cell confluency or stem-cell differentiation using spectroscopy-based ECIS [51].

Impedance analysis has also been widely used for the analysis of single cells or small tissue models by using electrodes, the size of which was comparable to that of the sample of interest. The liquid containing the samples was flown over or between the electrodes (coplanar or facing electrodes), a technique now known as impedance cytometry [52, 53], or confined in the sensing area, e.g., by hydrodynamic trapping [54] or physical barriers [24, 55–57].

To increase the signal-to-noise ratio, impedance cytometry is often carried out using a differential detection scheme by employing, e.g., a three-electrode setup [53]. Differential measurements enable to directly measure the dielectric properties of the sample against the suspension medium and to remove any effect caused by drifts in electrode performance, medium evaporation, or temperature variations. Owing to the relatively high throughput of impedance cytometry (~100-1000 cells per minute), this technique can be used to provide a snapshot of the sample condition at a defined time point and has been used to, e.g., identify cells in mixed cell populations [21, 58], recognize differentiated mesenchymal stem cells [59], measure the proportion of activated platelets in whole blood [60], and discriminate activated T-cells [61]. Conversely, sample confinement in the active area is a prerequisite to continuously monitor dynamic processes on the same sample or specimen. As an example, hydrodynamic trapping was employed to capture single yeast cells to follow yeast growth and budding by monitoring impedance variations over a large range of frequencies [54]. Impedance characterization has also been used to quantify the nonalcoholic fatty liver disease progression in 3D liver-microtissue models or to monitor the efficacy of candidate drug compounds *in vitro*, e.g., by recording the growth of cancer microtissues [57, 62] or the motility of human parasite larvae [24].

#### 2.3.2. Multielectrode Configurations

Two-dimensional “impedance imaging” of biological samples can be achieved by using a MEA, which features a matrix of independently addressable microelectrodes [63, 64]. As for the previously mentioned ECIS sensors, cell adherence to the microelectrode causes an increase in electrode impedance, which can be recorded by the 2D array electrodes to yield an image of cell dispersion and adherence over the array. The spatial resolution that can be attained with microelectrode arrays is defined by the electrode dimensions, the electrode pitch, and, in the z-direction, the ionic strength of the solution and the detection frequency [11]. Furthermore, both, electrode pitch and readout multiplexing capabilities, which define the spatial and temporal resolution, strongly depend on the technological approach used for the development of the MEA: *passive* MEAs with off-chip readout circuitry feature strong limitations in the number of electrodes that can be used in parallel and typically have additional space between the electrodes for routing and leads; *active* MEAs include addressing and signal-conditioning circuitry and are fabricated in complementary-metal-oxide-semiconductor (CMOS) technology; they feature large numbers of electrodes at a very small pitch and enable highly parallel impedance recordings from many electrodes. More details on the advantages and disadvantages of the two MEA categories will be discussed in the following sections.

**Figure 4:**
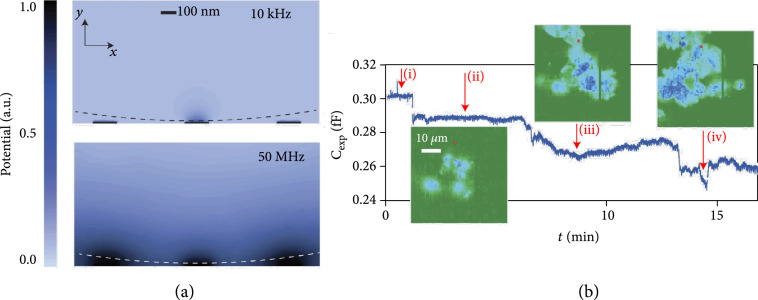
Impedance-imaging with HD-MEAs for the detection of cellular adhesion and micromotion. (a) Simulated spatial distribution of the electrical potential in 150 mM salt solution at 10 kHz and 50 MHz stimulation frequencies. The fields are simulated for a 90 nm diameter Au electrode and the presence of a 4.4 *μ*m polystyrene bead (dashed line) positioned on the array. At low frequencies, the electric field only interacts with the bead when the bead is located within ~10 nm distance from the electrode, due to the screening effect of the electrode-electrolyte double layer. By increasing the stimulation frequency to 50 MHz, the electric field extends further into the solution. (b) Capacitance values measured with a single-electrode (right), marked in red, during (i) PBS washing, (ii) introduction of cells in medium, (iii) cell attachment, and (iv) cell micromotions; adapted with permission from Laborde et al., Nat. Nanotech. 2015 (11).

Impedance-based detection with MEAs has been used to monitor the growth and distribution of biofilms on electrode arrays [65], to detect the hybridization of DNA strands that have been previously attached to the sensor surface [10, 66, 67], and to measure the dynamic attachment and micromotions of cells [11]. A notable example of using MEAs for real-time imaging of adherent cells is the work of Laborde et al., where the authors presented a CMOS high-density microelectrode array (HD-MEA) with 65,536 electrodes of 90 nm radius on a 0.6 μm×0.89 *μ*m grid [11]. To increase the detection range to a few hundreds of microns from the sensor surface, the authors used a 50 MHz detection frequency to overcome the screening effect of the electrical double layer (EDL) at the electrode interface (Figure 4(a)). While this approach enables to extend the sensing region of the electrodes while reducing the impedance of the double-layer capacitance at the sensing frequency, measuring only the capacitive contribution makes it impossible to perform impedance spectroscopy for cell characterization and differentiation. Nevertheless, the developed system enabled to follow, in real-time and at subcellular resolution, the adhesion, spreading, and dynamic attachment of cancer cells, which were cultured on the sensor surface (Figure 4(b)).

A primary use of MEAs includes localized voltage recordings, for which the systems have been used to perform long-term measurements of intra- and extracellular electrical potentials of electrogenic cells, in particular of neurons and cardiomyocytes *in vitro* [68–70]. With the realization of multifunctional electrode arrays, i.e., arrays of electrodes that can be utilized with multiple detection schemes (e.g., voltage, impedance, or electrochemical measurements), impedance-based imaging has been integrated in several systems to provide complementary information on the sample under investigation.

Chi et al. presented a sensor array featuring 144 independently addressable electrodes for extracellular voltage recording, impedance mapping, and optical detection, either via shadow imaging or bioluminescence [71]. The authors demonstrated the suitability of their system for the investigation of different cell types, namely, human cardiomyocytes, mouse neurons, and ovarian cancer cells. Through impedance imaging, the authors could monitor the cell attachment to the sensor array, while the integrated optical detection scheme provided information on the location and distribution of cells. Furthermore, the authors treated the cardiomyocytes with isoproterenol, a drug against bradycardia, and were able to detect an increase in the beating rate by using the integrated extracellular voltage recording. Cell attachment to the array was not affected by drug dosage, as could be detected in parallel by using impedance analysis. However, although the integrated optical and impedance-based capabilities provided an image of the sample fraction that was in direct contact with the electrode array, the spatial resolution was limited to ~90 *μ*m by the electrode pitch and by the nonuniform electrode distribution in the active sensing area.

**Figure 5:**
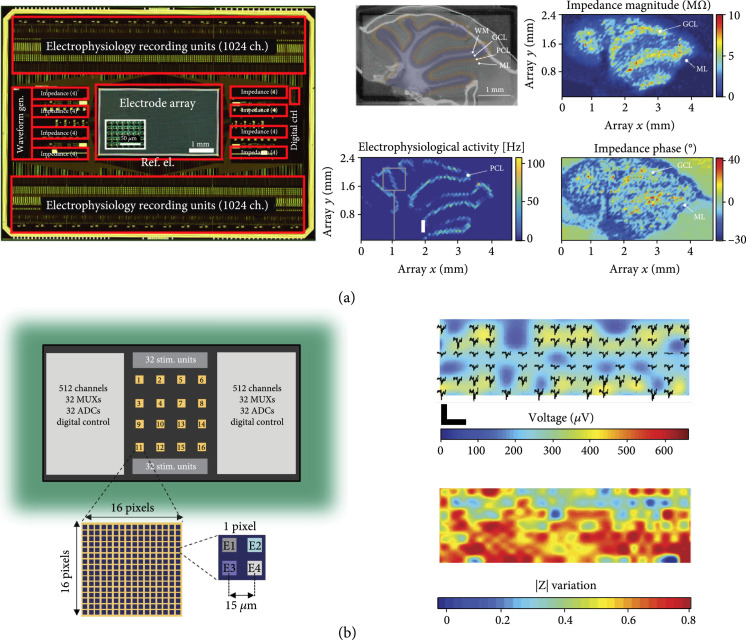
Multifunctional HD-MEAs. (a) A micrograph of the HD-MEA chip with the main functional blocks highlighted in the picture (left). The array featured 59,760 platinum electrodes at a 13.5 *μ*m pitch. The HD-MEA was used for impedance imaging and electrophysiological recordings of an acute brain slice (right). The electrical recordings are compared to a micrograph of the slice; adapted with permission from Viswam et al., IEEE Trans. Biomed. Circuits Syst. 2018 (12). (b) HD-MEA with 16 active areas, each of which includes 1024 TiN electrodes. The main functional blocks are indicated in the chip schematic, including 64 stimulation units and 1024 voltage-recording units, 64 multiplexers (MUX), and 64 analog-to-digital converters (ADC). Impedance recordings were used to reconstruct the spatial distribution of primary neurons that were cultured on chip and to correlate their positions with their electrophysiological activity (right); adapted with permission from Miccoli et al., Front. Neurosci. 2019 (13)–Creative Commons Attribution License (CC BY).

Our group presented a multifunctional HD-MEA, which featured 59,760 microelectrodes with an electrode pitch of 13.5 *μ*m (Figure 5(a)) [12, 44]. The system enabled to record impedance spectra with up to 32 electrodes in parallel in a frequency range between 1 Hz and 1 MHz. The small electrode pitch provided subcellular spatial resolution, for extracellular voltage recording and impedance measurements. The HD-MEA was used to monitor the differentiation of mouse embryoid bodies (EBs), which were plated on the array. Impedance imaging enabled to follow the adhesion, spreading, and growth of EBs on the array for five days. After five days of spreading and differentiation on chip, extracellular voltage recordings were used to measure the spontaneous beating of cells that had differentiated into cardiomyocytes [12]. The HD-MEA system was also used to acquire an impedance image of an acute mouse cerebellar slice and enabled to identify four different regions in the slice, namely, (i) white matter, (ii) the granular cell layer, (iii) the Purkinje cell layer with large electrophysiological activity, and (iv) the molecular layer that contained the dendritic trees of the Purkinje cells (Figure 5(a)). Impedance imaging enabled a label-free detection of different cell layers in the cerebellar slice, which could be recognized by their different impedance signatures (i.e., magnitude and phase). To perform a complete impedance scan of the whole sensing area, multiple sequential measurements were required due to the large number of active electrodes and the limited number of impedance-detection circuitry modules. Although recording at medium/high frequencies could be performed within a few minutes, low-frequency measurements required an acquisition time of ~1 hour, which is comparable to the time required for confocal imaging. Impedance imaging still offers the advantage to record in real time from live cell and tissue models, as there is no risk of potential damages by phototoxic effects during long-term optical and fluorescence imaging and as impedance imaging is label-free.

Lopez et al. recently presented a multifunctional HD-MEA featuring 16,384 electrodes, which were arranged in 16 active areas of 1024 electrodes each at an electrode pitch of 15 *μ*m [72]. The system featured current and voltage stimulation, intracellular and extracellular voltage recordings, and two modes for impedance measurement: fixed frequency (1 and 10 kHz) measurements for fast impedance monitoring (0.1-1 ms temporal resolution), and impedance spectroscopy in a frequency range between 10 Hz and 1 MHz. Impedance recordings at 1 kHz stimulation frequency were used to evaluate the adhesion and growth of primary hippocampal neurons on the chip (Figure 5(b)) [13]. The combination of voltage and impedance recordings provided information on cell distribution to assess whether the absence of electrical activity in specific regions on the sensor surface corresponded to areas devoid of cells or whether the regions were populated by cells without electrical activity. The impedance recording of the whole array at a single frequency could be acquired in ~2 minutes, while imaging of the whole sensor surface with a confocal microscope required ~30 minutes. The system enabled high spatial resolution within the 480×480 *μ*m^2^ sensing areas owing to the small electrode pitch of 15 *μ*m. However, the spatial arrangement of the electrode clusters, which featured a 1020 *μ*m intercluster distance, was optimized for multiwell packaging, which obviated to perform high-resolution electrical detection over large areas, e.g., for large tissue slices.

Finally, MEAs can be used to perform localized electrochemical measurements, such as amperometry and voltammetry, to provide real-time two-dimensional electrochemical imaging [44, 73–75]. As an example, a HD-MEA with 17.5×15 *μ*m^2^ interdigitated electrodes was used to detect the spatiotemporal characteristics of neurotransmitter release (norepinephrine, epinephrine, and dopamine) in acute murine adrenal-tissue slices upon stimulation with caffeine [75]. A microfluidic channel was used to achieve temporally defined chemical stimulation of the tissue slices so that the neurotransmitter release could be measured at high spatial and temporal resolution. Electrochemical measurements were also used to image the secretion of metabolites in bacterial films of *P. aeruginosa* [74]. Interestingly, the use of potential-sweep methods (square-wave voltammetry), as opposed to fixed-potential detection, enabled to detect the presence and concentration of multiple phenazine metabolites released by the bacterial film. This MEA allowed for monitoring the gradient distribution of metabolite secretion over a large sensing area of 8×8 mm^2^ at 225 *μ*m spatial resolution, which corresponds to the electrode pitch. Up to 38 electrodes could be read out in parallel, and each potential sweep was carried out in 0.2 seconds. Impedance imaging can be used to provide information that is complementary to functional analysis through voltage recordings or electrochemical imaging for an in-depth analysis of cell and tissue models *in vitro.*

Electrode impedance tomography (EIT) is a technique to generate 2D or 3D impedance-based images of a sample. EIT uses a set of electrodes, placed at specific locations of the sample, to inject small alternating currents and to measure the resulting voltages [76]. Currently, EIT is used in medicine and industry, for example, for label-free and continuous monitoring of patient respiratory parameters during mechanical ventilation [77, 78], while its use for *in vitro* biological applications has also been explored [76, 79–81].

A first example of EIT application in pharmaceutical research included its use to monitor the dissolution of pharmaceutical tablets [76]. 80 electrodes were integrated within a test vessel, and the dissolution of sodium chloride tablets in water was monitored in real time by detecting local variations in conductivity caused by the dissolved salt. EIT enabled to follow the dissolution process without interfering with the stirring of the solution during the test. However, the analysis imposed strict requirements on the sample type and experimental conditions: the dissolution experiment needs to be carried out in solutions with low conductivity, such as distilled or tap water, and sample dissolution needs to cause a local increase in conductivity, which restricts the analysis to salts or charged forms of drugs.

A prototype system featuring a cuboidal sample container, equipped with 18 electrodes on opposing faces for current injection and 360 voltage-sensing electrodes on three “imaging” sides, was proposed as a potential approach to monitoring tissue models *in vitro* [80]. The system was tested with different physical models of dimensions of several millimeters to assess the performance of the micro-EIT system and the reconstruction algorithms. These preliminary experimental tests were used for the optimization of micro-EIT test systems, which could theoretically provide a spatial resolution of less than 100 *μ*m for monitoring the growth of small tissue models in real time.

**Figure 6:**
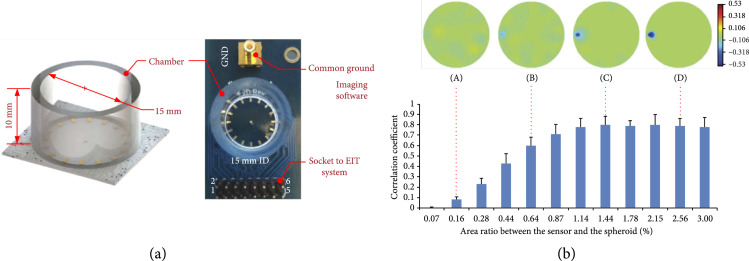
EIT sensor chip. (a) Schematics and picture of a planar EIT sensor at the bottom of a 15 mm diameter tissue-culture well. (b) The sensor enabled to reconstruct the 3D shape of microtissues of different size positioned within the well (top). The correlation between the EIT-reconstructed image and microscopy image for each microtissue size was reported (bottom); adapted with permission from Wu et al., Analyst 2018-Published by The Royal Society of Chemistry [79].

Imaging of breast cancer microtissues was achieved with a planar EIT sensor, which consisted of 16 microelectrodes, placed along the circumference of the base of a cylindrical cell-culture well of 15 mm in diameter, plus one grounded microelectrode at the center of the well (Figure 6) [81]. By exploiting a novel image reconstruction algorithm, the system was able to produce a 3D image representation of a microtissue spheroid of 550 *μ*m diameter, which corresponded to ~3.7% of the diameter of the EIT system. The same system was then used to monitor, in real time, the loss of cell viability of a cancer spheroid exposed to Triton-X, which is known to rapidly permeabilize the cell membrane [79]. New reconstruction algorithms based on machine learning have been recently proposed to further improve the spatial resolution of micro-EIT systems [82]. Finally, while EIT enables to provide information on the internal impedance distribution within 3D cellular constructs, the development of reconstruction algorithms for each application and setup is not trivial, which currently limits the wide adoption of this imaging technique.

## 3. Circuitry for Impedance Measurements: Circuit Topologies

Impedance sensing relies on measuring the relationship between the voltage drop across a sample and the current flowing through it. Different circuit topologies can be selected according to the most relevant requirements of the detection task, such as desired accuracy, frequency selectivity, measurement speed, parallelizability, or simplicity of the implementation.

MEAs can be assigned to two main categories: (i) *passive* electrode arrays, which feature metal electrodes on top of glass or silicon substrates that are then connected to external impedance measurement equipment; (ii) *active* electrode arrays, mostly fabricated in CMOS technology, which feature monolithic integration of electrode array and, at least, parts of the readout circuitry on the same chip. CMOS-MEAs offer the possibility to devise high-density electrode arrays with tens of thousands of electrodes that can be used for multiple functions [83, 84] and enable a high level of parallelization. In this section, we will review the main circuit topologies and implementations for impedance recording with MEAs and HD-MEAs.

### 3.1. Potentiostats

**Figure 7:**
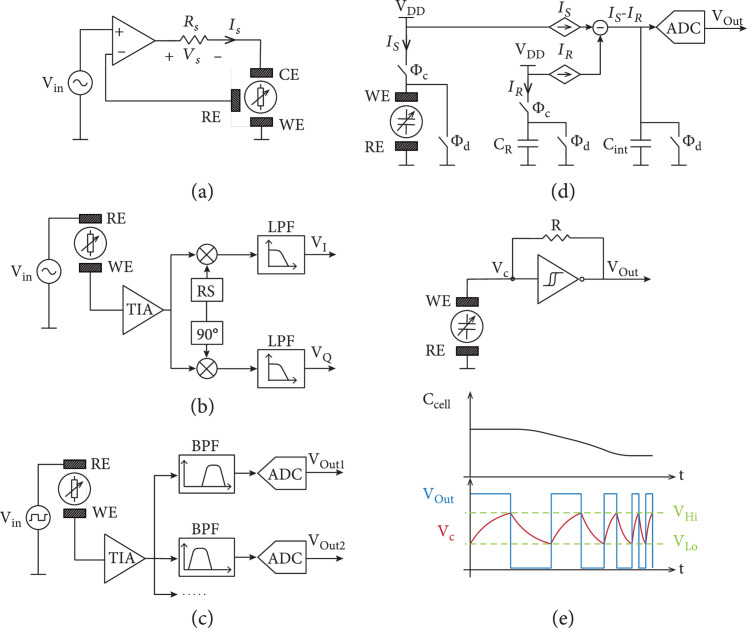
Simplified schematics of the circuit topologies commonly used for impedance sensing. (a) Potentiostat-based impedance sensing. The voltage difference between reference (RE) and working electrode (WE) is controlled by an operational amplifier, which injects a current through the counter electrode (CE). The injected current that is required to keep the sample voltage equal to the stimulation voltage ($V_{\text{in}}$) depends on the sample impedance. The injected current can be measured by using a known resistance ($R_{\text{s}}$) in series with the WE and by measuring the potential drop ($V_{\text{s}}$) across it. (b) Lock-in detection. A stimulation voltage is applied to the sample, and the resulting current is converted to a voltage using a TIA. The TIA output is multiplied with in-phase and quadrature-phase reference signals (RS) to demodulate the signal at the frequency of interest. The demodulated signals are then low-pass filtered to eliminate out-of-target-frequency noise and to calculate the signal amplitude and phase. (c) Simultaneous multiple-frequency detection. The applied stimulation signal can be a square pulse or a linear combination of multiple sinusoidal waveforms. The response of the sample is measured with a wide-range amplifier, which is then followed by multiple band-pass filters (BPFs), and digitized by a set of analog-to-digital converters (ADCs) in parallel to simultaneously measure the sample response at multiple frequencies. (d) Charge-based capacitive sensing. The sample capacitance and a reference capacitor ($C_{\text{R}}$) are charged to a constant voltage ($V_{\text{DD}}$) using two switches ($\Phi_{\text{c}}$), while measuring the current flowing through each capacitor ($I_{\text{S}}$ and $I_{\text{R}}$, respectively). The difference between these two currents is injected into a third capacitor, which acts as an integrator ($C_{\mathrm{int}}$). The resulting voltage is proportional to the difference between sample and reference capacitances. After digitizing the output voltage, all capacitors are discharged ($\Phi_{\text{d}}$). (e) Capacitance-to-frequency conversion based on a relaxation oscillator. The sample acts as a capacitor, which is repeatedly charged and discharged between two threshold voltages ($V_{\text{Lo}}$ and $V_{\text{Hi}}$) by a comparator with hysteresis and a feedback resistor (R). The oscillation period is proportional to the time constant of the resulting RC circuit, which depends on the sample capacitance.

The operation principle of a potentiostat is based on controlling the potential difference between a working and a reference electrode by applying a current through a counter electrode (Figure 7(a)) in a classical three-electrode setup. An operational amplifier with negative feedback is used to adjust the current and to counterbalance any deviation from the target voltage. Potentiostats are widely used for electrochemical measurements and in commercial impedance analyzer devices [85–88]. The sample impedance is calculated from the ratio between the applied voltage and the measured current, i.e., by using a known resistor in series to the sample.

Goikoetxea et al. designed an impedance measurement system, which featured a 20-channel, passive MEA with TiN electrodes [87], where the electrode impedance was monitored with a benchtop potentiostat by sequentially multiplexing between the electrodes. Zhang et al. presented a single-cell impedance measurement system with 128 passive electrodes that used dielectric forces to locate and drive the cells on top of the measurement electrodes [88]. Moreover, the authors used an external potentiostat-based impedance analyzer to carry out the impedance recordings.

Potentiostat-based detection is commonly used in commercial equipment owing to its wide frequency spectrum, high dynamic range, and suitability for electrochemical measurements. This detection method is not common in CMOS integrated circuits for impedance detection, as it typically requires high circuit complexity and large silicon area and potentially entails high power consumption, all features that ultimately limit parallelizability and large-scale integration.

### 3.2. Lock-In Amplifiers

A lock-in amplifier enables to extract low-amplitude signals with known periodicity from a noisy background and to measure single-frequency signals with remarkable accuracy. Lock-in detection is carried out by multiplying the readout signal with in-phase and quadrature (i.e., 90°-shifted) reference periodic signals, the periodicity of which equals that of the signal of interest. The multiplication results in a demodulation of the signal information at low frequencies, typically referred to as in-phase/quadrature (I/Q) demodulation, where undesired components and out-of-target-frequency noise can be easily eliminated using a low-pass filter. By multiplying with both, the in-phase and quadrature reference signals, lock-in detection enables to measure the real and imaginary component of the signal [89, 90]. The same periodic signal can be used for stimulation and for generation of the reference signals for lock-in demodulation, which enables to measure the absolute magnitude and phase delay of the detected signal (Figure 7(b)) [10]. Typically, a sinusoidal voltage stimulation is generated by the lock-in amplifier. The current flowing through the test system is converted into a voltage using a TIA before feeding it back to the lock-in detector for multiplication with the reference signals and subsequent low-pass filtering. Impedance values can then be extracted by calculating the ratio of the applied stimulation voltage and the measured current.

Lock-in detection is widely used in active CMOS-MEAs for obtaining impedance recordings of cells on electrode arrays. Chi et al. proposed an impedance sensing circuit for an integrated MEA system, where the reference signal was applied to one electrode and the sensing current was measured from four adjacent electrodes [71]. The sensed current was then multiplied with in-phase and quadrature reference current signals, low-pass filtered, and amplified with a variable-gain amplifier on chip. The low-pass-filtered and amplified signal was digitized off-chip using a data acquisition (DAQ) board, which enabled to record up to nine sensing units in parallel. Impedance signals were then extracted by calculating the ratio between the voltage signal applied at the reference electrode and the average current flowing through the four sensing electrodes. Modified versions of this impedance sensing circuit, all based on off-chip digitalization, have been presented by the same research group [91, 92]. However, off-chip digitalization increases the complexity of the experimental setup and strongly limits the number of electrodes that can be simultaneously recorded from.

Viswam et al. demonstrated an impedance spectroscopy system, based on an integrated lock-in amplifier. The on-chip waveform generator provided a sinusoidal voltage stimulation with a tunable frequency in a frequency range between 1 Hz and 1 MHz. The current flowing through the sensing electrode was then converted to a voltage via an integrated TIA, multiplied with the in-phase and quadrature reference signals, and subsequently, low-pass filtered and digitized on-chip using a sigma-delta analog-to-digital converter (ADC) [12, 44]. The system included 32 impedance measurement units in parallel, which could be connected to any of the 59,760 platinum electrodes of the array, to measure both the real and imaginary components of the impedance in parallel. However, the system only allowed to detect the impedance at a single frequency at a time for all connected electrodes. To perform impedance spectroscopy analysis, it is required to sweep the excitation signal across multiple frequencies sequentially, which resulted in long acquisition times per frequency sweep depending on the number of detected frequency points and the frequency range. A similar sequential approach was also used in the integrated HD-MEA, developed by Lopez et al. [72]. However, here, the authors used square-wave current-stimulation signals, while the cell impedance was measured by detecting voltages at the sensing electrodes. This impedance measurement structure takes advantage of current generators and amplifiers that were already implemented in the HD-MEA for cellular stimulation and voltage recording, which resulted in a compact and power-efficient design. Electrode voltage recording was performed by using an amplifier, equipped with choppers, which allowed to demodulate the impedance signal to low frequencies (<10 kHz) and to subsequently digitize those on-chip. The system featured 64 channels, which could be configured to provide either the in-phase or quadrature-phase signals so as to calculate the real and imaginary parts of the sample impedance off-chip. The spatial resolution of the system was limited by the multiplexed structure of the array, on which electrodes were arranged in pixels with four electrodes per pixel. While the electrode pitch was 15 *μ*m, only one of the four electrodes in each pixel could be recorded at a given time.

### 3.3. Simultaneous Multifrequency Detection

Analyzing the impedance at multiple frequencies using single-frequency lock-in detection can be time consuming, especially at low frequencies, as each frequency of interest must be measured sequentially. Therefore, different detection methods have been developed to enable the characterization of impedance across a wide range of frequencies at higher temporal resolution. To enable wideband detection, the stimulation signal must present a power spectrum, distributed across multiple frequencies, e.g., by using a pulse stimulation, a linear combination of several sinusoidal signals, or a random signal. Dedicated, specialized detection circuits have, therefore, been designed to be able to simultaneously process the system response across a wide range of frequencies of interest (Figure 7(c)).

Liu et al. demonstrated a pulse-based impedance-spectrum-measurement system using a four-electrode setup and off-chip components [93]. A short current impulse signal was applied to a cell using a pair of stimulation electrodes and amplified with a differential amplifier. The amplified signal was then band-pass filtered and digitized using a high-speed ADC. Impedance values across the spectrum of interest were then calculated by Fourier transformation of the digitized signal during postprocessing.

Bragos et al. proposed a multiple-frequency detection system that used broadband, burst stimulation signals, such as multisine waveforms with distributed frequencies, which were provided by an arbitrary-waveform generator [94]. The authors used a four-electrode configuration, with two current-stimulation electrodes and two voltage-readout electrodes. The front-end circuit included a voltage-controlled current source for generating the stimulation current, a differential amplifier for measuring the tissue-sample response, and a current-to-voltage converter for monitoring the applied stimulation current. All recorded signals were then captured by an external digital oscilloscope, and impedance calculation was then performed during data postprocessing. Although responses of the system at multiple frequencies could be obtained from a single measurement, postprocessing of the signals to extract the impedance values complicated data acquisition. Furthermore, since the stimulus signal contained power at multiple frequencies, the power spectral density at each frequency inevitably was low, which rendered the output signal more sensitive to noise. The accuracy of the measurement may be even more decreased during postprocessing due to the crosstalk between signals at different frequencies.

Hamilton et al. proposed a cell impedance sensor based on a “silicon cochlea” to provide simultaneous impedance sensing over a wide frequency range (from Hz to MHz) [95]. The simulated circuitry included both, a multiple-frequency signal generator (current stimulator) and an analyzer part. In this implementation, the voltage of a sensing electrode was fed to a voltage-to-current converter, and the corresponding current was split up into components at different frequencies with cascaded low-pass filters. However, the multiple analog outputs of the circuit require digitalization for each frequency output of the electrodes, which massively increases the complexity of the detection system.

Simultaneous multifrequency detection has not been widely adopted for integrated CMOS systems, probably due to the lower accuracy in comparison to well-established single-frequency techniques such as I/Q demodulation. However, simultaneous multifrequency detection may be suitable for applications where fast impedance monitoring has priority over high accuracy [72].

### 3.4. Capacitive Sensing

For many applications, measuring only the imaginary component of the impedance may be sufficient for extracting the information of interest, such as the presence of a cell on an electrode or cell adhesion [96]. Typically, capacitive measurements can be carried out using simpler circuit schemes than those used for absolute impedance detection. Figure 7(d) shows an example of a charge-based capacitive measurement (CBCM) circuit to measure variations of the electrode capacitance. First, the sensing electrode capacitance ($C_{\text{S}}$) and reference capacitance ($C_{\text{R}}$) are charged (through $\Phi_{\text{c}}$) to a fixed voltage, while the charging currents are sensed. The reference current ($I_{\text{R}}$) is then subtracted from the sensing current ($I_{\text{S}}$), and the difference is accumulated in the integrating capacitor ($C_{\mathrm{int}}$). The resulting voltage is afterwards digitized with an ADC and provided as the output. Finally, the system is reset by discharging (through $\Phi_{\text{d}}$) the capacitors.

Nabovati et al. developed an 8×8 MEA, where the capacitance difference between sensing and reference electrodes was measured by using the CBCM system as illustrated in Figure 7(d) [97, 98]. The output voltage was then digitized with an on-chip sigma-delta ADC and was analyzed to estimate the capacitance change during postprocessing. Despite featuring a comparably large electrode pitch (~180 *μ*m), which limited spatial resolution, the fully integrated MEA enabled to detect small capacitive changes caused by single cells growing atop the array owing to the interdigitated electrode structure. A much higher spatial resolution was obtained by Widdershoven et al., who developed a HD-MEA of 65,536 gold-copper nanocapacitor electrodes for high-frequency impedance imaging [11, 99]. The readout circuit used the CBCM technique to observe the capacitance change on the electrodes and to obtain real-time capacitance images of cells. Finally, another example of capacitive sensing for impedance analysis was described by Prakash et al. [100]. The authors presented a charge-sharing-based capacitive sensor for tracking cell adhesion and cell health. The authors could differentiate healthy and diseased cells by detecting the differences in cell adherence to the electrode surface. The stronger adherence of healthy cells led to a higher capacitive coupling at the electrode, which could be detected by charging and discharging the electrode capacitance.

These examples show how capacitive coupling can be used to obtain high-resolution electrical imaging of cells on electrode arrays and information on cell adherence. However, the use of high-frequency detection limits the amount of information that can be extracted with this technique.

### 3.5. Impedance-to-Frequency Conversion

With the previous methods, impedance is quantified by measuring the current through (or voltage across) a sample to which a stimulus is applied. However, impedance can also be estimated by integrating the sample as part of an electrical oscillator, so that the oscillator frequency is directly dependent on the sample impedance. Measuring oscillation frequencies can be carried out in a relatively simple way, especially with modern CMOS technology, where hundreds of oscillators and frequency-measurement units can be combined into the same integrated circuit. Therefore, this detection method is of great interest for achieving massively parallelized measurements. The topology of the oscillator and the type of impedance to be analyzed directly influence the oscillator sensitivity (i.e., the relationship between oscillation frequency and impedance) and phase noise (i.e., random frequency fluctuations), which dictate the fundamental limit of accuracy of this technique [101, 102].

Relaxation oscillators are a common choice for capacitive sensors, since the oscillation is generated by charging and discharging a capacitor between two voltage thresholds (Figure 7(e)). Van der Goes and Meijer presented a readout circuit based on a relaxation oscillator that has been sequentially connected to a capacitive sensor and fixed capacitors [45]. An off-chip microcontroller was used to measure the oscillation frequencies, and the sample capacitance was then calculated from the differences in oscillation frequency. The use of fixed capacitors as reference enabled a continuous autocalibration of the readout circuit, rendering this solution more robust against undesired drifts caused by fluctuations in environmental conditions or against fabrication process variations. However, the use of an off-chip microcontroller for frequency measurement impedes parallelization and the integration of multiple units in an array. Stagni et al. presented a DNA sensor array featuring 128 oscillator-based, differential capacitive sensors with on-chip counters for the parallelized monitoring of the respective oscillation frequencies [66].

## 4. Design Considerations for Different Applications

In addition to circuit topology for sensing, several other factors need to be considered for the design of a MEA or HD-MEA system for impedance detection. These factors include the geometry and material of the electrodes, the MEA topology, signal digitalization, and multiplexing approaches, as well as sensitivity and noise characteristics. The different design options will be discussed in the following sections. We then summarize a variety of representative solutions that have been reported in literature in a table which compares different approaches to designing MEA or HD-MEA systems (Table 1).

**Table 1:** Applications, circuitry architectures, and design options of some representative MEA-based impedance measurement systems.

	Application	Frequency range (Hz)	Number of electrodes	Electrode size	Electrode material	Electrode pitch (*μ*m)	Circuitry architecture	MEA type
Liu et al. 2009 [120]	Detection of cell adhesion, spreading, and proliferation	1-1 M	100	80 *μ*m${}^{*}$	Pt	200	Potentiostat-based sensing	Passive
Mucha et al. 2011 [121]	Detection of cell reactions and adhesion	—	64	55×55 *μ*m^2^	Au	—	Impedance-to-frequency converter	Active
Mamouni et al. 2011 [122]	Detection of cell adhesion, spreading, and proliferation	10-1 M	50	15×15 *μ*m^2†^	Au	—	Potentiostat-based sensing	Passive
Widdershoven et al. 2015 [99]	Impedance imaging of cells, detection of dynamic attachment, and cellular micromotion	1 M–70 M	65,536	180 nm${}^{*}$	Au	0.6×0.89	Capacitive sensing	Active
Dragas et al. 2018 [44]	Detection of cell adhesion, differentiation, and spreading, impedance imaging of tissue slices	1-1 M	59,760	3×7.5 *μ*m^2^	Pt black	13.5	Lock-in detection	Active
Lopez et al. 2018 [72]	Detection of cell contractility with impedance monitoring module, detection of cell morphology, differentiation, and adhesion with impedance spectroscopy module	10–1 M^‡^	16,384	2.5×3.5 *μ*m^2^ 4.5×4.5 *μ*m^2^ 6.5×7 *μ*m^2^ 10.5×11 *μ*m^2^	TiN	15	Lock-in detection	Active
Goikoetxea et al. 2018 [87]	Characterization of biofilm structure	1-100 k	64	60 *μ*m${}^{*}$	TiN	100	Potentiostat-based sensing	Passive
Jung et al. 2019 [92]	Detection of cell distribution, growth, and proliferation	15 k-500 k	21,952	8×8 *μ*m^2^	Au	16	Lock-in detection	Active
Nabovati et al. 2019 [98]	Detection of cell-surface binding	1-100 k	64	5×25 *μ*m^2†^	PEM^§^	~180	Capacitive sensing	Active

∗Diameter of circular electrodes. ^†^Interdigitated electrodes. ^‡^1 Hz and 10 kHz for fast impedance monitoring at fixed frequency. ^§^Polyelectrolyte multilayer films.

### 4.1. Electrode Geometries

Electrode size, the surface-area ratio between reference and sensing electrodes, and the electrode pitch are key parameters that directly affect the signal quality, detection sensitivity, and lateral resolution.

Cells sedimenting on or attachment to an electrode causes an increase of the electrode impedance, as they block the electric currents through fractions of the electrode surface. The measured impedance increases as the ratio between the electrode area and cell dimension decreases [103]. Generally, a decrease in electrode size or area results in an increase of the electrode impedance as a consequence of the smaller surface area and the smaller electrical double-layer capacitance at the electrode-electrolyte interface [50]. The effects of a high initial impedance of small electrodes may superimpose to or even obscure cell-dependent variations at low frequencies, in particular, when the electrode diameter is below ~50 *μ*m. For such small electrodes, the electrode impedance in physiological solutions can be in the M*Ω* range for frequencies up to ~10 kHz, if no surface modification is applied to increase the effective electrode area [104]. The selection of a suitable electrode size strongly depends on the application of interest. For example, electrodes of hundreds of microns in diameter can be employed for monitoring the formation of a confluent cell layer [7, 50]; such large electrodes feature a large sensing area and allow for using simplified readout circuitry schemes, however strongly affecting the spatial resolution and signal sensitivity attainable.

The electrode size and pitch directly define the spatial resolution that can be attained in impedance imaging. The development of integrated MEAs towards HD-MEAs featuring large numbers of small electrodes with dimensions comparable to the size of cells at low pitch enabled them to attain more detailed information including tissue morphology and spreading [11].

Impedance imaging is usually carried out in conjunction with other types of measurements, such as voltage measurements, so that an electrode size needs to be found that yields sufficient sensitivity and acceptable noise characteristics for all implemented detection methods. Simply decreasing electrode size and increasing the electrode number to improve spatial resolution results in complex readout architectures to handle the large number of electrodes or increased measurement time in case of a limited number of readout units, as well as higher microfabrication costs.

Moreover, the counter electrode in three-electrode setups or the reference electrode in two-electrode setups has to be much larger than the sensing or working electrodes to provide low-impedance paths for current injection [50]. In MEA architectures that use alternating array electrodes as counter/reference electrodes for each sensing electrode, the size of the counter/reference electrode is defined by the array electrode size and pitch. However, for MEA architectures featuring shared reference electrodes, the reference electrode size can be chosen with more freedom, as such shared electrodes are usually placed outside the array so that their size is not limited by the size and pitch of working or array electrodes. Finally, the distance between sensing and reference electrodes also affects impedance measurements [50, 103]. If working and counter or reference electrodes are placed too close to each other, the current directly flows between the electrodes without being affected by cells or biological samples so that the measured impedance only depends on electrode and medium resistance [105, 106].

### 4.2. Electrode Materials

Electrodes for impedance measurements of biological samples have been realized with different materials, such as gold (Au), platinum (Pt), silver/silver chloride (Ag/AgCl), titanium nitride (TiN), iridium oxide (IrO_2_), or ultrananocrystalline diamond [2, 44, 87, 107]. Gold and platinum are common material choices for MEA fabrication, as these materials feature high biocompatibility and stability and low resistivity, and they are compatible with microelectronic postprocessing, which is a fundamental requirement for the realization of highly integrated HD-MEA systems. Au and Pt surface properties can be readily altered by surface modification to, e.g., increase the effective electrode surface area by increasing surface roughness, which leads to a reduction of the electrode impedance by orders of magnitude [104, 108]. Examples include the deposition of gold nanoparticles and carbon nanotubes on gold microelectrodes [108] or the electrodeposition of platinum black (Ptb) on platinum electrodes [104]. Surface modification imposes an additional step in the fabrication process, and to achieve a uniform deposition across the whole electrode array can be challenging. Surface properties can vary among electrodes or deposition runs, and characterization of individual electrode properties is required to compare measurements of different sensors. Finally, porous TiN coatings have also been used for HD-MEAs in *in vitro* and *in vivo* applications, as this material has shown electrical performance comparable to that of Au and Pt and high stability under physiological conditions [13, 109]. A recent trend towards the use of 3D *in vitro* models has also fueled the development of 3D-MEA structures. Metal-based electrodes (typically Au and Pt) can be fabricated on different micro- and nanostructures, such as flexible polyimide pillars [110] or silicon-based shanks [111], to realize 3D electrode arrays, which then can be interfaced with standard, planar readout circuitry.

Metal materials, however, are nontransparent and prevent optical access to the sample with inverted microscopes, which represents a major limitation for standard *in vitro* investigations of biological samples. Therefore, transparent, conductive materials, such as indium tin oxide (ITO) or functionalized iridium oxide (IrO_2_), have been explored as alternative electrode materials for passive MEAs on glass wafers to enable simultaneous optical and electrical characterization of cells *in vitro* [112–115].

Recently, the use of hydrogel-based electrodes, which more closely match the mechanical and physical properties of biological samples, has been proposed [116]. Hydrogel-based electrodes have not yet been widely applied in MEAs; however, the interest in integrating hydrogel electrodes is rapidly growing, also for potential use in implants *in vivo* [117]. Finally, new fabrication approaches, such as silicon nanowires [118] or multilayered metal nanostructures [70], have been reported in literature to realize nanometer-dimension 3D electrodes that enable highly parallel intracellular recordings without damaging cellular structures.

### 4.3. Electrode Array Topologies

MEA systems include conductive electrodes and the readout circuitry to record, amplify, and filter the signals. In passive MEAs, electrodes are fabricated on a solid substrate (usually glass or silicon) [119] and connected via leads and wires to external readout circuitry. In contrast, active or integrated MEAs or HD-MEAs are usually monolithic systems, including microelectrodes and readout circuitry on the same silicon substrate and taking advantage of the high level of integration provided by CMOS technology. The design and fabrication of passive MEAs are simple, fast, and considerably less expensive than that of integrated MEAs or HD-MEAs, which facilitates rapid prototyping and optimization of electrode size and shape for different applications. However, the long connections between electrodes and readout circuits in passive MEAs typically entail higher noise levels, and the number and density of electrodes in the array are limited. In contrast, long and noisy connections are avoided in active MEA solutions, as the readout circuits are on the same substrate and at very short distance (typically less than a few millimeters) from the sensing electrodes, which results in better noise performance [84]. Furthermore, reading from tens of thousands of (sub-)micron size electrodes at low pitch, as they are available in modern HD-MEAs, requires dedicated on-chip addressing, signal processing, and sampling or multiplexing schemes that can be fabricated with state-of-the-art CMOS technologies [11–13]. Therefore, the availability of CMOS HD-MEA technology is key for the realization of impedance-imaging systems, as the spatiotemporal resolution of the imaging is determined by electrode numbers and size, electrode pitch, and the achievable data acquisition rate.

### 4.4. Signal Conditioning, Digitalization, and Multiplexing

As mentioned above, signal conditioning, multiplexing, and digitalization strategies form part of fundamental design considerations for CMOS HD-MEA-based impedance sensors. Signal conditioning typically includes amplification and filtering to transform weak and noisy signals, detected at the electrodes, into robust signals for processing in subsequent stages. The design of the amplifiers has to be done with due regard to the expected input signals and the dynamic range and resolution in subsequent stages. The gain must be large enough to ensure that the smallest features of interest remain detectable during further processing. However, extremely large gains may cause system saturation in the presence of large input signals. To be able to record input signals with different amplitude ranges, amplifiers can feature variable gain, which can be adjusted with respect to the input signal amplitude [12, 72]. For measurements based on current sensing, signal conditioning stages may include a TIA for current-to-voltage conversion, since voltage signals are typically preferred for processing in subsequent stages. Analog lock-in amplifiers can be integrated as part of the signal conditioning chain, including the demodulation of high-frequency signals at low frequencies, which eases further analog processing [10, 12]. Besides amplification, filtering is performed to attenuate out-of-band signals. High-pass filtering is only required, if the expected input signal has low-frequency (or continuous) components that would saturate amplifiers or adapt baselines between different stages. However, low-pass filtering is required in most designs to limit the signal bandwidth before sampling and to prevent aliasing in the next stages.

Different multiplexing and digitalization schemes impose different requirements on the performance of the different components of the readout chain, such as noise characteristics, bandwidth, and available area for analog processing units as well as the number, dynamic range, speed, and resolution of the ADCs. A first option is to condition and digitize the signal directly at the sensing site, followed by multiplexing of the digital signals [66]. This implementation provides the possibility of performing parallel measurements with a large number of electrodes. However, the performance of the sensing and digitalization circuits is limited by the available area in each electrode pixel, which, in turn, determines electrode density.

An alternative approach includes to perform simple signal conditioning at each sensing electrode and to multiplex the resulting signals to a set of ADCs outside the active area for signal digitalization, which disentangles the ADC requirements and electrode pixel area (electrode size and pitch) [72]. This alternative implementation requires a lower number of ADCs, and signal processing (including lock-in detection) can be carried out digitally on chip or during off-chip postprocessing. The challenge here lies in the design of high-performance ADCs, both in terms of resolution and speed, to avoid losing information during signal digitalization and multiplexing. However, complex signal processing, such as signal filtering and multiplication, can be implemented in the analog domain and the digitalization of the processed signal can be done afterwards [12]. In such an approach, the design challenges mostly concern the design of analog processing circuits, however, with the benefit of relaxed requirements for the design of the ADCs.

### 4.5. Sensitivity and Noise

The detection limit of a sensor is determined by its signal-to-noise ratio (SNR), which defines the minimum signal that can be reliably detected given the measurement noise. Here, we refer to measurement noise as any fluctuation in the recorded signal that is not considered a signal, i.e., is caused by a change in the sample. High SNR requires high sensitivity, i.e., large variations of the output signal upon small variations in the sample, and low noise levels, i.e., small signal variations that are not related to changes in the sample impedance.

Maximizing the sensitivity of impedance measurements requires to find an optimum measurement frequency where a maximum change in signal output is obtained in dependence of impedance magnitude and/or phase changes of the sample. Simulations and analysis of sample impedance models can be used to determine detection frequencies, at which sample variations produce large impedance variations. As the effects of the parasitic elements and connection lines are often not included in the simulations, detection frequencies need to be determined experimentally [50, 103, 105]. Finally, sensor sensitivity is highly dependent on electrode size and surface properties. Therefore, the electrode properties, in particular the impedance of the electrode and its connections, need to be optimized to ensure good sensitivity in the frequency range of interest [97].

The most efficient approaches to reduce signal noise rely on reducing the intrinsic noise of the read-out components, filtering out the out-of-band noise, and using differential measurement configurations. Noise can be introduced at any stage of the signal generation and readout, i.e., during stimulation, signal amplification, demodulation, and filtering, as well as during data conversion and transmission. For the impedance measurement methods that are based on applying a stimulus and recording the resulting current/voltage, such as lock-in detection or the use of potentiostats, the SNR can be improved by increasing the amplitude of the applied stimulus to the highest possible levels at which the sample is not affected and the input range of the readout circuits is not exceeded.

Frequency-domain filtering is an effective way to reduce the noise by removing out-of-band signal contributions, since specific sample features may only be detected within defined frequency ranges. Ideally, the filter should feature a very narrow passband around the frequencies of interest for the impedance measurement. The design of such filters can be challenging in the analog domain, so signals are frequently postprocessed in the digital domain. However, filtering is remarkably effective and simple for impedance sensors based on lock-in detection, since filtering of the downmodulated signal after multiplication can be carried out using a dedicated low-pass filter [12]. Finally, differential schemes, whether applied during signal acquisition or postprocessing, are an effective means to greatly reduce the effect of power-supply noise or environmental noise. Acquisition of differential signals, however, requires more circuitry, therefore larger areas for implementation, which could impact electrode density or increase fabrication costs. While differential values can often be calculated during data postprocessing, this solution may yield lower accuracy and less noise suppression than differential signal acquisition.

## 5. Outlook

Impedance imaging is a real-time, label-free, and noninvasive measurement method, which can be used for long-term characterization of cells and tissue models. The small form factors of the sensor chips and the electronic nature of the detection render this technique particularly suitable for parallelization and automation. Real-time monitoring of cell and tissue dynamics can be performed very efficiently and rapidly in comparison to optical microscopy. High sampling rates, which can be achieved by measuring the sample impedance at a single high frequency, enable to detect rapid dynamics, such as cell micromotions, on the sensor surface at high spatiotemporal resolution and over large sensing areas (up to few mm^2^). Although EIS imaging may feature a low level of specificity in the discrimination of different cell types, which only depends on the dielectric properties of the cells, impedance-based imaging enables to characterize sample optical access to which is not possible, such as tissue slices on MEAs during electrophysiological recordings or brain tissue *in vivo* while using MEA-based brain implants [123–125]. The development of MEA brain implants has been mainly focused on the recording of neural activity or brain stimulation. However, the presence of hundreds or thousands of implanted electrodes would also enable to study the dielectric properties of the environment in which the devices were implanted and to perform imaging under conditions of no optical access. Detecting variations in the surroundings of the implants could potentially be useful to track the position of electrodes or to monitor electrode surface properties and the integrity of the host tissue over longer periods of time.

Further developments of MEAs or HD-MEAs for impedance imaging revolve around increasing the number of electrodes and reducing their pitch to achieve high spatial resolution. CMOS-based HD-MEAs represent the only viable approach to attain (sub)micrometer resolution and highly parallel detection, although the integration of thousands of compact and power-efficient circuits represents a major design challenge. As advances in CMOS technology have greatly improved the efficiency of digital circuits in comparison to their analog counterparts, an early digitalization of signals and a minimization of analog processing on chip may provide a viable solution. At the same time, increasing the number of measurement units will produce large datasets that need to be recorded and processed, which requires fast data-acquisition systems and powerful data-analysis units, especially in case of multifrequency measurements. Therefore, digitalization and signal preprocessing, such as Fast Fourier Transformations (FFTs), directly on-chip will likely play a key role in the development of highly parallelized HD-MEAs as they offer the possibility to rapidly acquire impedance recordings over a large spectral range at high temporal resolution. However, the development of CMOS-MEAs requires—owing to design complexity and fabrication costs of integrated circuits—much larger time and financial investments in comparison to the fabrication of passive MEAs. Therefore, the development of custom-designed CMOS-MEAs for specific applications can only be justified by either unique functionality or performance that cannot be achieved otherwise or by large numbers of devices that can be produced at comparably low costs.

Finally, technological and theoretical improvements in electrical impedance tomography (EIT) may also increase the relevance of impedance techniques for studying biological samples. Multifrequency impedance tomography (also known as electrical impedance tomography spectroscopy; EITS) is a promising noninvasive technique to analyze frequency-dependent characteristics of a sample [126]. The complexity of the computations required to reconstruct the image of the sample requires novel reconstruction algorithms and efficient hardware implementations. Machine learning techniques may prove useful for handling and simplifying the high-dimensional data required through EIT [127, 128], especially for imaging approaches involving multifrequency measurements. The resolution and robustness of EIT can also be improved by including *a priori* knowledge about the sample and adjusting image reconstruction algorithms to exclude reconstructed images that are not compatible with the known physical properties of the sample [129, 130]. Passive electrode arrays with low numbers of electrodes can be controlled through field-programmable gate arrays (FPGAs), on which efficient postprocessing algorithms can be implemented [131, 132]. CMOS HD-MEAs are well suited for performing EIT measurements owing to their small pitch and large number of electrodes. However, given that CMOS HD-MEAs are prevailingly implemented in older technologies (such as 0.18 *μ*m), the performance of on-chip digital circuitry is poor in comparison to modern nanometer-technology CMOS processors. Therefore, it may be more efficient to perform image reconstruction using separate dedicated electronic chips.
